# Mapping of Candidate Genes for Nitrogen Uptake and Utilization in *Japonica* Rice at Seedling Stage

**DOI:** 10.3390/genes15030327

**Published:** 2024-03-02

**Authors:** Ning Chen, Tianze Ma, Sijia Xia, Chengxin Li, Yinuo Liu, Jiaqi Wang, Guize Qu, Hualong Liu, Hongliang Zheng, Luomiao Yang, Detang Zou, Jingguo Wang, Wei Xin

**Affiliations:** 1 College of Agriculture, Northeast Agricultural University, Harbin 150030, China; neau_chenning@163.com (N.C.); 17843095455@163.com (T.M.); xiasijia_duoduo@163.com (S.X.); l1184061160@163.com (Y.L.); 19991176292@163.com (J.W.); qu2215123581@163.com (G.Q.); liuhualongneau@163.com (H.L.); zhenghongliang008@163.com (H.Z.); yaochang616@163.com (L.Y.); zoudtneau@126.com (D.Z.); 2 Harbin Academy of Agricultural Sciences, Harbin 150030, China; 15022169475@163.com; 3 Key Laboratory of Germplasm Enhancement and Physiology & Ecology of Food Crop in Cold Region, Ministry of Education, Northeast Agricultural University, Harbin 150030, China

**Keywords:** rice (*Oryza sativa* L.), GWAS, RNA-seq, QTLs, nitrogen

## Abstract

Nitrogen (N) is one of the essential nutrients for the growth and development of crops. The adequate application of N not only increases the yield of crops but also improves the quality of agricultural products, but the excessive application of N can cause many adverse effects on ecology and the environment. In this study, genome-wide association analysis (GWAS) was performed under low- and high-N conditions based on 788,396 SNPs and phenotypic traits relevant to N uptake and utilization (N content and N accumulation). A total of 75 QTLs were obtained using GWAS, which contained 811 genes. Of 811 genes, 281 genes showed different haplotypes, and 40 genes had significant phenotypic differences among different haplotypes. Of these 40 genes, 5 differentially expressed genes (*Os01g0159250*, *Os02g0618200*, *Os02g0618400*, *Os02g0630300*, and *Os06g0619000*) were finally identified as the more valuable candidate genes based on the transcriptome data sequenced from Longjing31 (low-N-tolerant variety) and Songjing 10 (low-N-sensitive variety) under low- and high-N treatments. These new findings enrich the genetic resources for N uptake and utilization in rice, as well as lay a theoretical foundation for improving the efficiency of N uptake and utilization in rice.

## 1. Introduction

Rice originated in tropical and subtropical regions and is one of the most important food crops in the world [1]. Since the beginning of the 20th century, the continuous increase in fertilizer, especially nitrogen (N) fertilizer, has made a great contribution to the improvement of crop yields worldwide [2]. The moderate application of N fertilizer can increase yields and improve economic efficiency, but farmers tend to apply excess N fertilizer in crop production [3,4]. This tendency has led to significant reductions in N uptake and utilization and also caused a number of ecological problems [5], as well as the aggravation of crop failure, pests, and disease [6,7]. Therefore, reducing the amount of N fertilizer, improving the uptake and utilization of N fertilizer, and ensuring high yields have become the main issue that rice breeders need to study.

In recent years, there have been plenty of studies on N uptake and utilization in plants [8,9,10,11]. The N-efficient genotype in cotton, CCRI-69, showed higher N concentration and N accumulation at both N levels [12]. *TabZIP60* is a negative regulator of wheat growth and N utilization. The overexpression of *TabZIP60* can inhibit wheat growth, while reducing *TabZIP60* expression by RNAi interference can improve wheat yield and N utilization efficiency (NUE) partially; the expression of NADH-dependent glutamate synthase (NADH-GOGAT) was up-regulated by RNAi interference so as to improve wheat yield and NUE [13]. *OsLHT1* is responsible for the uptake of aspartate, and the reduction in N uptake and utilization by knocking down *OsLHT1* led to growth inhibition and yield reduction in rice [14]. *OsNLP1* regulates nitrate and ammonium utilization by coordinating multiple genes for N uptake and utilization. One study showed that the transcription of *OsNLP1* was influenced by the N environment, and the overexpression of *OsNLP1* can improve yield and NUE in rice under different N conditions [15]. *Indica* rice plants carrying the *DNR1* allele showed a decrease in *DNR1* nitrogen response transcription and protein abundance, which, in turn, positively regulated growth hormone synthesis, thereby activating the growth hormone response factor-mediated NO_3_^−^ transporter (*OsARF*), which further promoted NUE and seed yield by inversely activating N-metabolism-related genes [16]. These studies are of great importance for understanding the molecular mechanisms of N uptake and utilization in rice. However, the related genes and their specific molecular mechanisms need to be further explored.

Genome-wide association analysis (GWAS) has been widely used to study complex traits in rice and to locate their associated quantitative trait loci, such as cold tolerance [17], salt tolerance [18], starch content [19], grain length [20], thousand-grain weight [21], etc. There have been many studies on N uptake and utilization, such as the identification of seven NUE-related genes (including *OsNPF6.1* and *OsNAC42*) using a natural population consisting of 117 rice varieties with extreme N-related phenotypes [22]. *OsTCP19* was identified through the GWAS of 110 rice varieties at similar growth stages and the analysis of their tillering responses to N, which showed that *OsTCP19* served as a modulator of the tillering response through its transcriptional response to N and its targeting of the tiller-promoting gene DWARF AND LOW-TILLERING [23]. *OsNLP4*, a NUE-related NODULE INCEPTION (NIN)-like protein gene, was identified by GWAS using rice core germplasm [24]. The transcriptome has also been widely used in studies mining genes related to N utilization in rice. For example, after using 0.1 mM (NH_4_)_2_SO_4_ to treat N-deficient rice seedlings for 4 h and 12 h, the shoots and roots were taken for RNA-seq, and the final analysis demonstrated that JA and flavonoids are important signaling molecules in response to low NH_4_^+^ in roots [25]. An integrated analysis of the transcriptome, metabolome, and co-expression network identified 15 candidate genes that coordinate carbon metabolism, N metabolism, and N effectiveness [26]. The integration of GWAS and transcriptome data has also been used to analyze the genetic mechanisms of many complex traits. For example, three approaches, linkage analysis, association analysis, and transcriptome analysis, were used to complement each other and validate eight genes associated with high-temperature tolerance in rice [27]; the integration of GWAS and transcriptome data identified 2 candidate genes associated with bacterial wilt [28] and 13 Cd-related genes in rice [29]. However, GWAS for low-N tolerance using cold-region *japonica* rice germplasm is relatively rare.

In this study, the N content and N accumulation in the leaves and roots of 295 *japonica* rice varieties were measured under low- and high-N treatments at the seedling stage. GWAS was performed on the phenotypic values of the above traits and 788,396 SNPs to identify QTLs associated with low-N stress. Then, a haplotype analysis of the genes within the QTL intervals was conducted to obtain candidate genes for low-N tolerance. The candidate genes were further screened by transcriptome analysis, and the final candidate genes for low-N tolerance were obtained. The results of this study provide a theoretical basis for illustrating the molecular mechanism of N uptake and utilization in rice.

## 2. Materials and Methods

### 2.1. Plant Material and Genotyping

The panel of the natural population is composed of 295 *japonica* rice germplasms collected mainly from China, Korea, Japan, and Russia. The DNA of 295 *japonica* rice varieties was extracted, and high-throughput sequencing was performed using an Illumina HiSeq 2000. SNPs were detected using the GATK4.2.1.0 software toolkit [30], and a total of 788,396 SNPs were obtained for GWAS analysis by removing SNPs with the lowest allele frequencies, below 0.05, and deletion rates greater than 20% in the initially obtained population SNPs [31]. The genetic structure of the population was calculated using ADMIXTURE1.3.0 software [32]. The genetic structure of this population, among others, was demonstrated in a pre-publication article from our lab [31].

### 2.2. Phenotypic Data

Two hundred plump seeds from each of the 295 *japonica* rice germplasms were treated in a 48 °C oven for 48 h to break dormancy. Then, the seeds were sterilized with a 0.01% sodium hypochlorite solution for 30 min, washed with sterile water three times to remove the sterilization solution residue from the seed surface, placed in an incubator at 31 °C, and incubated in the dark for 2 days. After germination, 64 germinating seeds of each variety with a uniform shoot length were selected and divided equally into two parts. One part would be treated with low N, and the other part would be treated with high N. The germinated seeds were placed in 96-well PCR plates, and one seed was placed in each well. The PCR plates with germinated seeds were then placed in plastic boxes and transferred to a greenhouse to grow seedlings (23.8 °C/22.4 °C, 10 h during the day/14 h at night). Two N fertilization treatments, low N (8 ppm) and high N (40 ppm) [33,34], were set up using NH_4_NO_3_ as the N source. All germinated seeds were first incubated under high N for 5 days (64 grains per variety). Then, 32 seeds of each variety continued to grow in a high-N solution for 21 days, and the other 32 seeds per variety were cultured in a low-N solution for the same 21 days [33]. The hydroponic nutrient solution was prepared by referring to the conventional formula specified by IRRI. The nutrient solution was replaced every 7 days. After 21 days of incubation under the two different N treatments, the relevant indexes were measured.

A total of 295 seedlings of *japonica* rice obtained after low- and high-N treatments were randomly taken from three plants each. The leaves and roots of the rice seedlings obtained after low- and high-N treatments were cut off, washed with ultrapure water, killed in an oven at 105 °C for 30 min, and dried in a constant-temperature oven at 80 °C for 12 h to a constant weight, and then the dry matter weight of the leaves and roots was weighed. The dried samples were put into 2 mL centrifuge tubes. Three to four 3 mm steel balls were added to each tube. Then, the samples were ground into powder using a sample-grinding machine. About 0.05 g of powder was weighed. The weight of the weighed sample was recorded accurately. The N content of the sample was determined using an elemental analyzer (Primacs SNC 100-IC-E), and N accumulation was calculated. Three replicates were set up for the above treatments, and the traits’ relative values were calculated.

N accumulation (mg/plant) = Leaf (Root) N content (%) × Leaf (Root) dry weight (mg).

Relative character value = Low-N treatment character value/High-N treatment character value.

### 2.3. GWAS

GWAS was performed using the MLM method in Tassel 5.0 software [35]. The GWAS of the phenotypes and SNP genotypes was performed based on the population structure (Q matrix) and the calculated genetic relationship between any two individuals (Kinship matrix). The threshold for SNPs significantly associated with the traits was set at *p* < 5.46 × 10^−6^, determined by genetic type 1 error calculator (GEC; http://statgenpro.psychiatry.hku.hk/gec/, accessed on 10 May 2023), which calculates the effective number of independent markers [36]. Manhattan plots and Q-Q plots were drawn for the association analysis results using the CMplot package in the R language. If two or more significant SNPs were located in the same linkage disequilibrium interval, these SNPs were considered the same QTL, and the SNP with the lowest *p* value was defined as the lead SNP of this QTL. To identify the lead SNP with the lowest *p* value, redundant SNPs were filtered at the minimum distance interval. The annotation information of genes within the QTL interval was checked against the Ensembl genome database.

### 2.4. Linkage Disequilibrium (LD) Analysis

The paired R2 values between any two SNPs within the ±2 Mb interval of the leading SNP were calculated using LDBlockShow [37]. The average of the top 10% paired R2 values in each interval was calculated and recorded as the background value of LD attenuation. The value obtained by adding 0.2 to the background value was considered the attenuation interval of LD.

### 2.5. Haplotype Analysis

The location interval of each gene was identified according to the China Rice Data Center (https://www.ricedata.cn/, accessed on 23 June 2023). Non-synonymous mutant SNPs within the exonic region of the gene were extracted using the RiceSNP SeekDatabase website (https://snp-seek.irri.org/, accessed on 23 June 2023) and used to identify the haplotype together with SNPs within the range of the first 2 kb of the start codon.

### 2.6. RNA-seq

Two comparison groups (H71 vs. L71 and H284 vs. L284) were set up. Specifically, 71 is Longjing 31 (a low-N-tolerant rice variety), 284 is Songjing 10 (a low-N-sensitive variety), and H and L represent high N and low N, respectively. Firstly, sixteen seedlings each of Longjing 31 and Songjing 10 were cultured under the high-N treatment (40 ppm) for 5 days; then, eight seedlings were kept in the same solution for 7 days, while the other eight seedlings were transplanted into the low-N treatment (8 ppm) for 7 days. After 12 days, 12 root samples (three replicates of each treatment) were collected. Total RNA was extracted from the 12 samples using the TransZol Up RNA Kit (TransGen Biotech, Beijing, China). Complementary DNA was synthesized from total RNA using the HiFiScript cDNA Synthesis Kit (CWBio, Beijing, China). An Illumina library was constructed according to the manufacturer’s instructions (Illumina, San Diego, CA, USA). High-throughput RNA sequencing was performed using the Illumina HiSeq 2500 platform. HISAT v2.1.0 was adopted to construct the index and to map clean reads to the reference genome. The rice reference genome data used was Os-Nipponbare-Reference-IRGSP-1.0. The gene alignment and FPKM (Fragments Per Kilobase of transcript per Million fragments mapped) were calculated by using featureCounts v1.6.2 [38]. *p* < 0.05 and |log_2_FC| > 0.585 were adopted as the thresholds to identify the differentially expressed genes between any two comparative groups using edgeR v3.24.3 [39]. Other specific experimental methods and transcriptomic data are shown in previously published articles from our laboratory [33].

### 2.7. Candidate Gene Prediction

All genes within the LD attenuation interval of every lead SNP in the GWAS results were integrated for candidate gene prediction; the phenotypic data of the 295 *japonica* rice varieties were combined with the SNP data to analyze all genes by haplotype, and the genes with significant phenotypic differences among different haplotypes among these genes were identified as candidate genes. Then, the genes with significant phenotypic differences among different haplotypes and |log_2_FC| > 0.585, *p* < 0.05 in the low-N-tolerant variety, Longjing 31, were screened as more valuable candidate genes by combining the gene expression from RNA-seq.

### 2.8. Quantitative Real-Time PCR

A qRT-PCR assay was performed using the same total RNA used for the transcriptome data analysis of the two comparison groups mentioned above (H71 vs. L71 and H284 vs. L284). The first-strand cDNA (10 μL) was synthesized according to the instructions for the PrimeScript™ RT Master Mix (Takara Biomedical Technology (Beijing) Co., Ltd., Beijing, China). Primer5.0 was used to design specific primers for this assay (Table S1). The BlazeTaqTM SYBR Green qPCR Master Mix 2.0 (GeneCopoeia, Guangzhou, China) was used, and reactions were run on Roche Lightcycler 96 real-time PCR equipment in accordance with the manufacturer’s instructions (Roche Medical Instruments, Basel, Switzerland). Each sample had three technical replicates, and *Actian1* was used as an internal control. Finally, the expression of 5 candidate genes was calculated using the 2^−∆∆CT^ method.

### 2.9. Data Analysis

The density distribution of each trait was plotted using “ggplot2” in the R4.2.3. The phenotypic data were analyzed using IBM SPSS Statistics 25.0 software (SPSS Inc., Chicago, IL, USA) for correlation analysis and descriptive statistics, including the mean, extreme deviation, and coefficient of variation.

## 3. Results

### 3.1. Phenotypic Data Analysis

**Figure 1:**
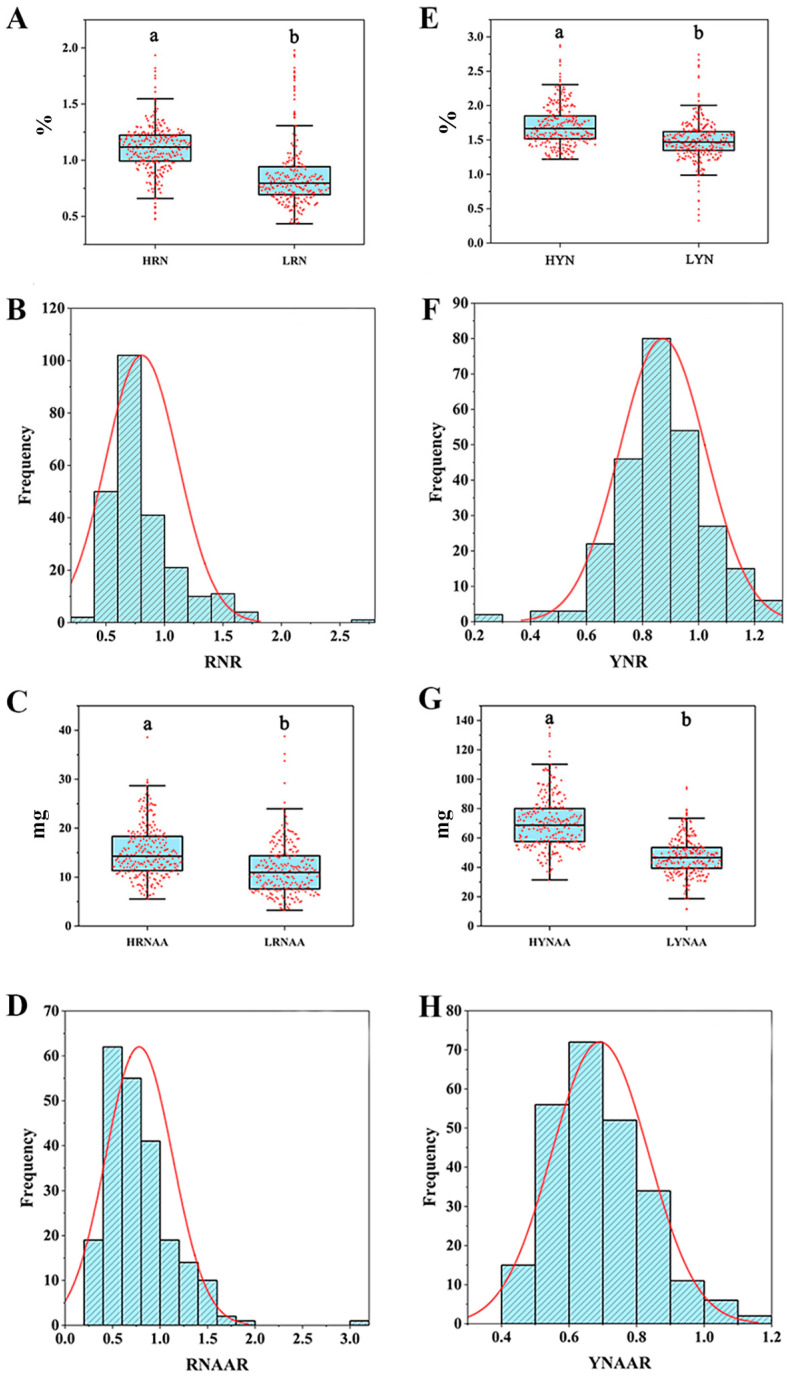
Phenotypes of 295 japonica rice varieties under high- and low-N treatments. (**A**) HRN (root N content under high-N treatment); LRN (root N content under low-N treatment). (**B**) RNR (relative value of root N content under low- and high-N treatments). (**C**) HRNAA (root N accumulation under high-N treatment); LRNAA (root N accumulation under low-N treatment). (**D**) RNAAR (relative value of root N accumulation under low- and high-N treatments). (**E**) HYN (leaf N content under high-N treatment); LYN (leaf N content under low-N treatment). (**F**) YNR (relative value of leaf N content under low- and high-N treatments). (**G**) HYNAA (leaf N accumulation under high-N treatment); LYNAA (leaf N accumulation under low-N treatment). (**H**) YNAAR (relative value of leaf N accumulation under low- and high-N treatments). Lowercase a and Lowercase b represent the significance between treatments at the 0.05 level.

The coefficients of variation of leaf N content (YN), leaf N accumulation (YNAA), root N concentration (RN), and root N accumulation (RNAA) of 295 *japonica* rice germplasm seedlings ranged from 20.76% to 46.25% and 17.43% to 35.20% under the high- and low-N treatments, respectively (Table S2). The coefficients of variation of the relative value of root N accumulation (RNAAR) and the relative value of leaf N content (YNR) were the largest and smallest under the low- and high-N treatments, respectively. The coefficients of variation of RN and RNAA under the low-N treatment were larger than those under the high-N treatment, while YN and YNAA were not significantly different between high- and low-N treatments. The phenotypic values of all traits showed abundant variation and were approximately normally distributed (Figure 1).

Under the low-N condition, all traits showed very significant positive correlations, with correlation coefficients ranging from 0.239 to 0.797, except for the significant positive correlation between YNAA and RN. Under the high-N condition, YN and YNAA, RN and RNAA, and YNAA and RNAA showed very significant positive correlations, with correlation coefficients of 0.338, 0.647, and 0.532, respectively. In relative values, YN and YNAA, RN and RNAA, and YNAA and RNAA showed very significant positive correlations, of which the correlation coefficients are 0.534, 0.861, and 0.287, respectively. There were very significant positive correlations between YN and YNAA, RN and RNAA, and YNAA and RNAA, whose correlation coefficients are 0.534, 0.861, and 0.287, respectively (Table S3).

### 3.2. QTL Localization

**Figure 2:**
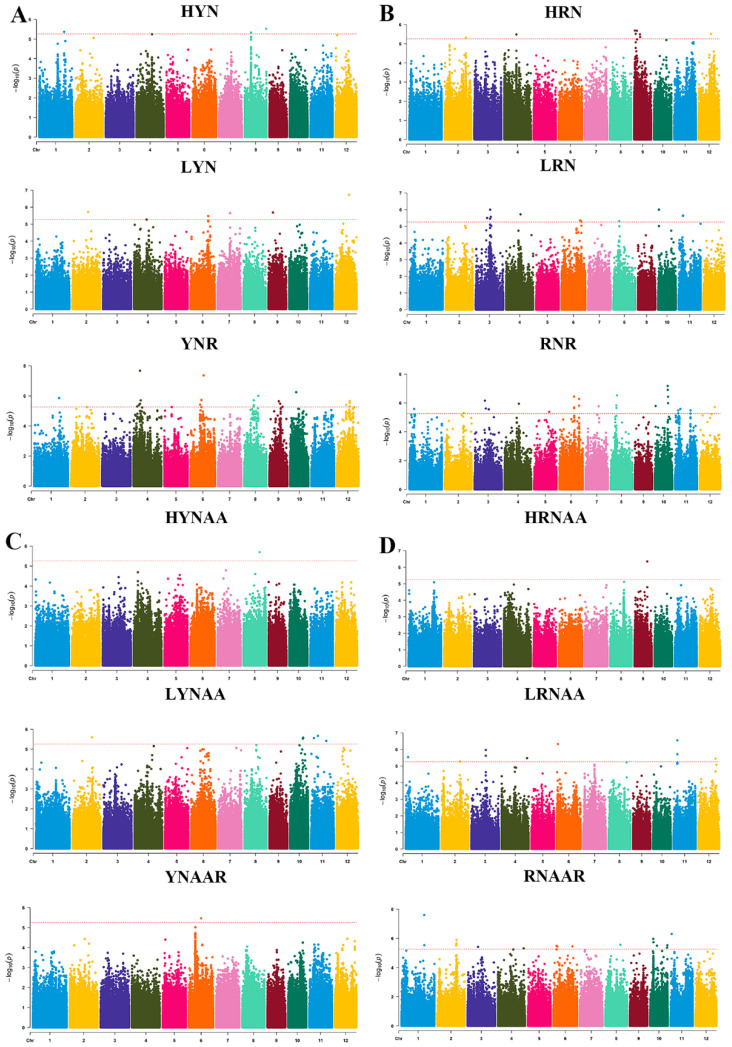
Manhattan plots of N content and N accumulation. (**A**) Leaf N content under high- and low-N treatments. (**B**) Root N content under high- and low-N treatments. (**C**) Leaf N accumulation under high- and low-N treatments. (**D**) Root N accumulation under high- and low-N treatments. The detailed annotations for abbreviations are shown in Figure 1.

A total of 75 SNPs that were significantly associated with traits related to N uptake and utilization in rice seedlings were localized (Table S4 and Figure 2). Among them, three SNPs located in the first and eighth linkage groups were significantly associated with HYN, and the corresponding QTLs were named qHYN1, qHYN8-1, and qHYN8-2, of which the phenotypic contributions were 9.79%, 9.84%, and 10.07%, respectively. The six SNPs significantly associated with LYN were located in the 2nd, 4th, 6th, 7th, 9th, and 12th linkage groups, and their corresponding QTLs were named qLYN2, qLYN4, qLYN6, qLYN7, qLYN9, and qLYN12, with phenotypic contributions ranging from 9.43% to 13.12%. The thirteen SNPs significantly associated with YNR were mapped to the 1st, 4th, 6th, 8th, 9th, 10th, and 12th linkage groups, and the corresponding QTLs were named qYNR1, qYNR4-1, qYNR4-2, qYNR4-3, qYNR6-1, qYNR6-2, qYNR7, qYNR8-1, qYNR8-2, qYNR9-1, qYNR9-2, qYNR10, qYNR12-1, and qYNR12-2, with phenotypic contributions ranging from 9.41% to 13.72%. The five SNPs significantly associated with HRN were located in the 2nd, 4th, 9th, and 12th linkage groups, and their corresponding QTLs were named qHRN2, qHRN4, qHRN9-1, qHRN9-2, and qHRN12, with phenotypic contributions ranging from 9.34% to 10.16%. The seven SNPs significantly associated with LRN were located in the 3rd, 4th, 6th, 8th, 10th, and 11th linkage groups, and their corresponding QTLs were named qLRN3-1, qLRN3-2, qLRN4, qLRN6, qLRN8, qLRN10, and qLRN11, with phenotypic contributions ranging from 9.34% to 10.63%. The sixteen SNPs significantly associated with RNR were located in the 1st, 2nd, 3rd, 4th, 5th, 6th, 7th, 8th, 10th, 11th, and 12th linkage groups, and their corresponding QTLs were named qRNR1, qRNR2, qRNR3-1, qRNR3-2, qRNR4, qRNR5, qRNR6-1, qRNR6-2, qRNR7, qRNR8, qRNR10-1, qRNR10-2, qRNR11-1, qRNR11-2, qRNR11-3, and qRNR12, with phenotypic contributions ranging from 9.68% to 13.33%. One SNP significantly associated with HYNAA was mapped to the eighth linkage group, and the corresponding QTL was named qHYNAA8, whose phenotypic contribution was 8.95%. Four SNPs significantly associated with LYNAA were located in the 2nd, 10th, and 11th linkage groups, and the QTLs were named qLYNAA2, qLYNAA10, qLYNAA11-1, and qLYNAA11-2, respectively, with phenotypic contributions ranging from 9.68% to 10.12%. One SNP significantly associated with YNAAR was identified in the sixth linkage group, which was named qYNAAR6, with a phenotypic contribution of 10.71%. One SNP significantly associated with HRNAA was identified in the 9th linkage group, and the corresponding QTL was named qHRNAA9, of which the phenotypic contribution was 11.67%. The seven SNPs significantly associated with LRNAA were located in the 1st, 2nd, 3rd, 4th, 6th, 11th, and 12th linkage groups, and the corresponding QTLs were named qLRNAA1, qLRNAA2, qLRNAA3, qLRNAA4, qLRNAA6, qLRNAA11, and qLRNAA12, respectively, with phenotypic contributions ranging from 10.21% to 12.46%. The eleven SNPs significantly associated with RNAAR were located in the 1st, 2nd, 3rd, 4th, 6th, 8th, 10th, and 11th linkage groups, and the corresponding QTLs were named qRNAAR1, qRNAAR2, qRNAAR3, qRNAAR4, qRNAAR6-1, qRNAAR6-2, qRNAAR8, qRNAAR10-1, qRNAAR10-2, qRNAAR10-3, and qRNAAR11, with phenotypic contributions ranging from 9.55% to 16.15%. Among them, Chr2__25361672 (qRNR2, qRNAAR2), Chr3__13879513 (qRNA3-1, qRNAAR3), Chr3__19077080 (qLRN3-2, qLRNAA3), Chr4__18968355 (qLRN4, qRNR4), and Chr10__889424 (qLRN10, qRNR10-2) were significantly associated with several different traits.

### 3.3. Candidate Gene Mining

The 75 QTL intervals contained 811 genes (Table S5). Among these genes, 232, 39, 8, and 2 genes had two, three, four, and five haplotypes among 295 *japonica* rice germplasms, respectively. Among the 281 genes with different haplotypes, 40 genes that had different haplotypes showed significant phenotypic differences in different traits among the 295 *japonica* rice germplasms. Therefore, it is hypothesized that these 40 genes might be associated with N uptake and utilization.

**Figure 3:**
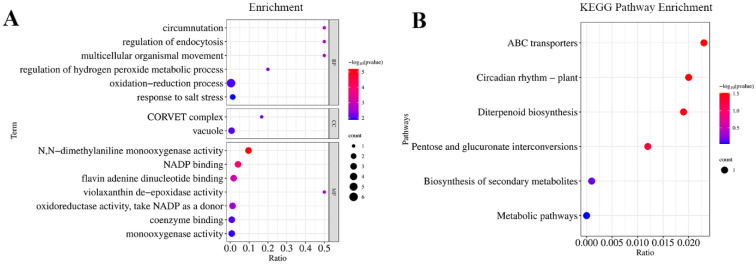
Enrichment analysis of 40 genes. (**A**) Top 15 categories from GO classification analysis of 40 genes. (**B**) KEGG functional classification and biological pathway enrichment of 40 genes.

A GO enrichment analysis of these 40 genes with significant haplotype differences showed that they could be enriched in 39 functional groups, including 21 biological processes, 3 cellular components, and 15 molecular functions. Circumnutation, regulation of endocytosis, multicellular organismal movement, N,N-dimethylaniline monooxygenase activity, NADP binding, flavin adenine dinucleotide binding, and Violaxanthin de-epoxidase activity were significantly enriched (Figure 3A). A KEGG enrichment analysis of these 40 candidate genes was also performed, and the results showed that a total of six pathways were enriched (Figure 3B).

### 3.4. Expression Analysis of Candidate Genes

**Figure 4:**
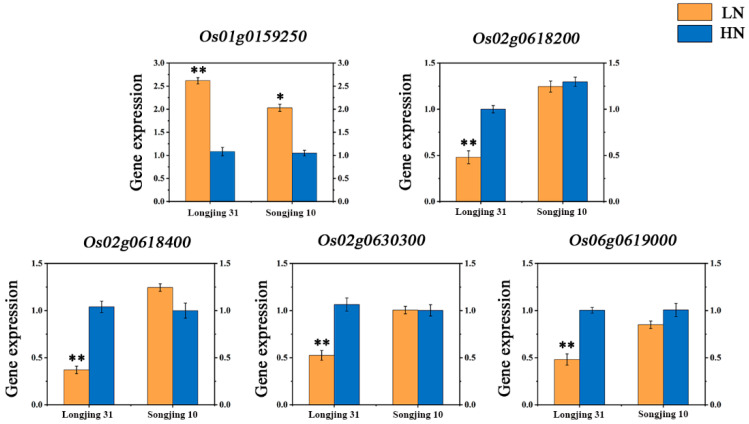
Gene expression of five candidate genes in Longjing 31 and Songjing 10 under high- and low-N treatments. * denotes significant difference at the 0.05 level, ** denotes significant difference at the 0.01 level.

**Figure 5:**
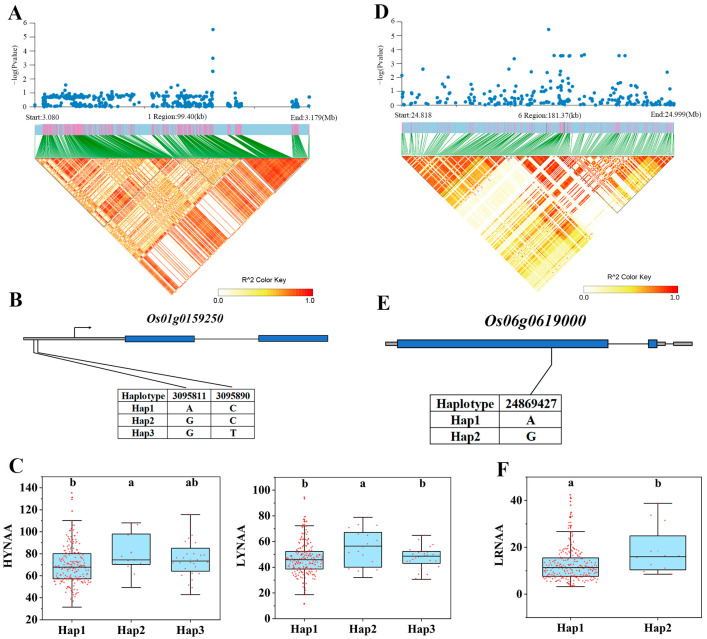
Associated region and haplotype analysis of *Os01g0159250* and *Os06g0619000*. (**A**) Regional Manhattan plot and linkage disequilibrium (LD) heatmap of *Os01g0159250*. (**B**) Gene structure of *Os01g0159250*. (**C**) Haplotype analysis of *Os01g0159250*. (**D**) Regional Manhattan plot and LD heatmap of *Os06g0619000*. (**E**) Gene structure of *Os06g0619000*. (**F**) Haplotype analysis of *Os06g0619000*. Lowercase a and Lowercase b represent the significance between treatments at the 0.05 level.

**Figure 6:**
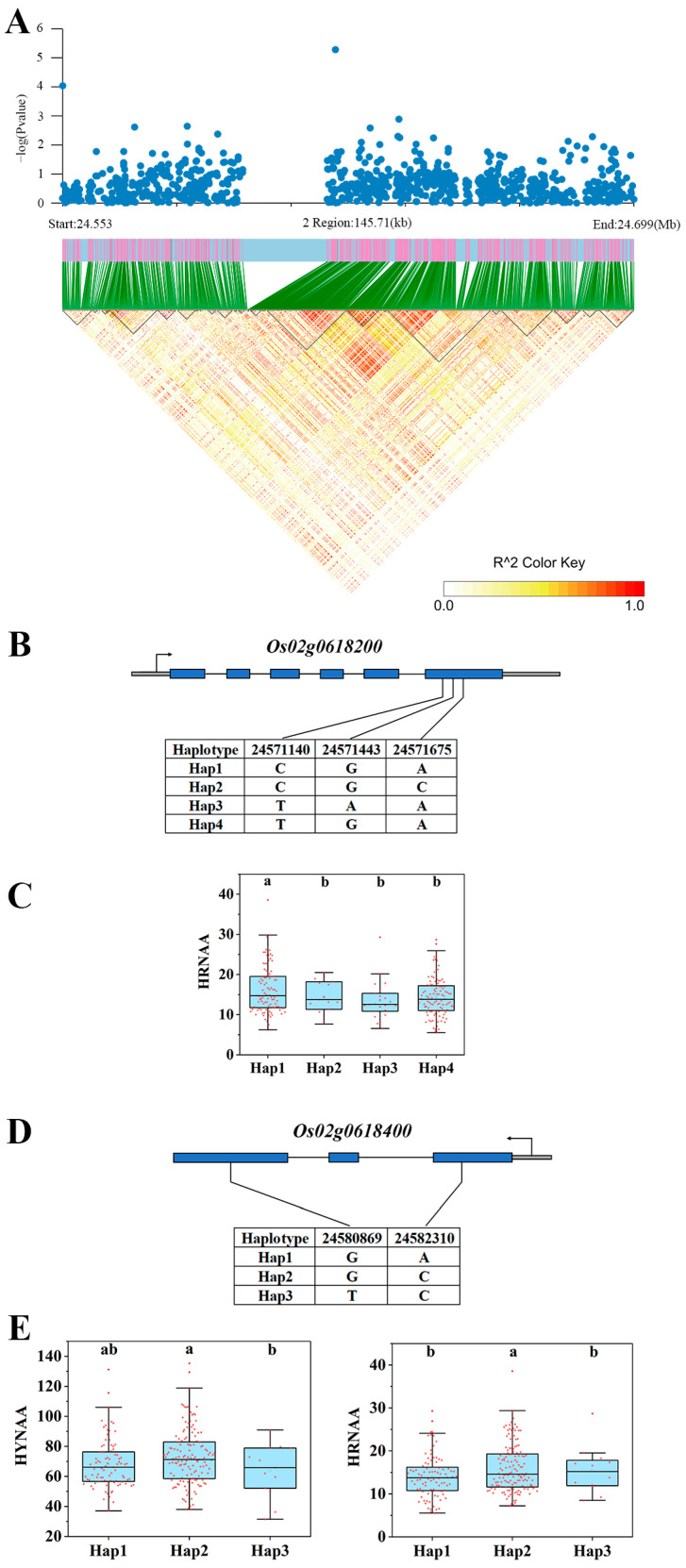
Associated region and haplotype analysis of *Os02g0618200* and *Os02g0618400*. (**A**) Regional Manhattan plot and linkage disequilibrium heatmap of *Os02g0618200* and *Os02g0618400*. (**B**) Gene structure of *Os02g0618200*. (**C**) Haplotype analysis of *Os02g0618200*. (**D**) Gene structure of *Os02g0618400*. (**E**) Haplotype analysis of *Os02g0618400*. Lowercase a and Lowercase b represent the significance between treatments at the 0.05 level.

**Figure 7:**
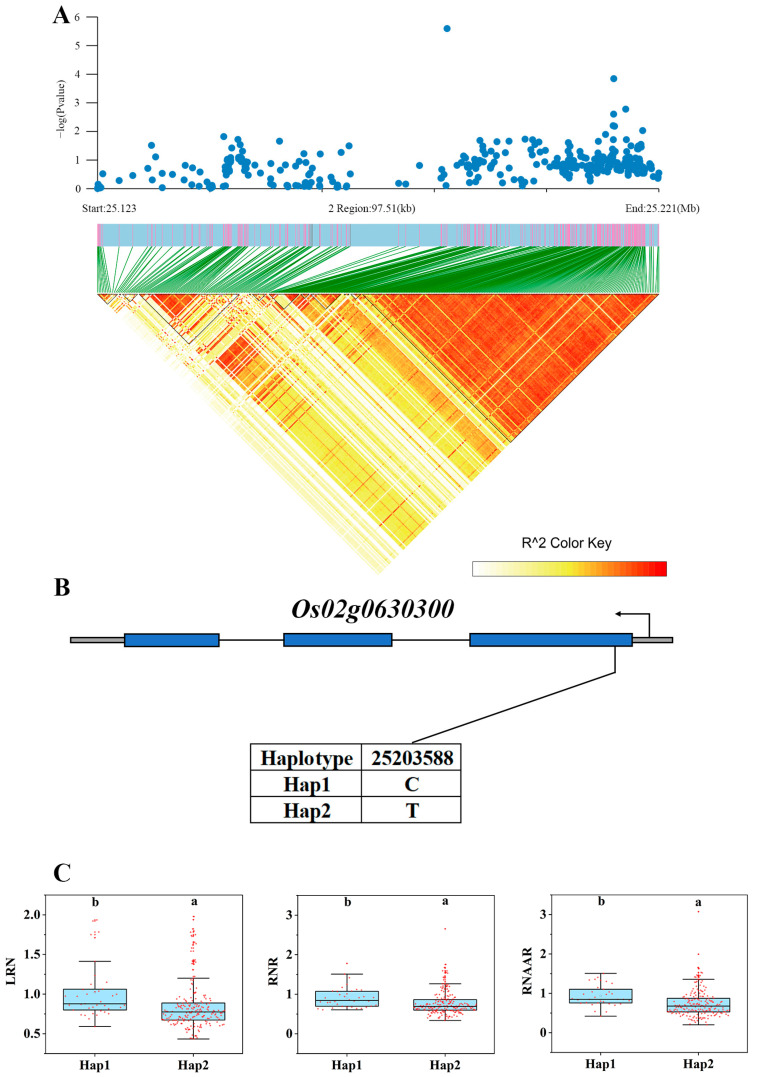
Associated region and haplotype analysis of *Os02g0630300*. (**A**) Regional Manhattan plot and linkage disequilibrium heatmap of *Os02g0630300*. (**B**) Gene structure of *Os02g0630300*. (**C**) Haplotype analysis of *Os02g0630300*. Lowercase a and Lowercase b represent the significance between treatments at the 0.05 level.

Transcriptome analysis was performed to elaborate on the expression differences of these 40 genes (Table S6). There were four significantly down-regulated genes and one significantly up-regulated gene in Longjing 31 (low-N-tolerant variety) and two significantly up-regulated genes in Songjing 10 (low-N-sensitive variety) between the treatment (low N) and control (high N). Finally, five genes, *Os01g0159250*, *Os02g0618200*, *Os02g0618400*, *Os02g0630300*, and *Os06g0619000*, which were differentially expressed in the low-N-tolerant variety, were selected as the most valuable candidate genes in this study (Table 1). The expression levels of these five candidate genes in Longjing 31 and Songjing 10 under low- and high-N treatments were verified by qRT-PCR. The qRT-PCR results of these five candidate genes were consistent with the expression trends of RNA-seq data (Figure 4). The linkage disequilibria of these six candidate genes are illustrated in Figure 5A,D, Figure 6A and Figure 7A, respectively.

### 3.5. Haplotype Analysis of Candidate Genes

Among the 295 japonica rice germplasms, two non-synonymous SNPs were present in the promoter region of *Os01g0159250*, forming three different haplotypes (Figure 5B). The three haplotypes showed significant phenotypic differences that existed in two traits, HYNAA and LYNAA (Figure 5C). *Os02g0618200* and *Os02g0618400* were located in the same QTL interval, and both of them had non-synonymous SNPs present in the CDS region. The number of non-synonymous SNPs and haplotypes of the two genes was three and two and four and three, respectively (Figure 6B,D). Four different haplotypes of *Os02g0618200* showed significant phenotypic differences in HRNAA (Figure 6C), while three different haplotypes of *Os02g0618400* showed significant phenotypic differences in HYNAA and HRNAA (Figure 6E). Both *Os02g0630300* and *Os06g0619000* had only one non-synonymous SNP in the CDS region and thus had two different haplotypes (Figure 5E and Figure 7B). Two haplotypes of *Os02g0630300* showed significant phenotypic differences in three traits, LRN, RNR, and RNAAR (Figure 7C). Two haplotypes of *Os06g0619000* showed significant phenotypic differences in HYNAA, LYNAA, and LRNAA (Figure 5F).

## 4. Discussion

Among all fertilizers, N fertilizer has been used as a necessary fertilizer to increase crop yields, whereas the excessive use of N fertilizer has caused many adverse effects. For example, the excessive application of N fertilizer has led to the significant accumulation of nitrate-N content in the 2–4 cm soil layer, causing groundwater and surface water hazards, which has become a widespread global problem [43]; the natural transformation of N in the soil reduces the pH of the soil and destroys the soil health [44]. Studies have shown an exponential increase in the coastal dead zones of the world’s oceans and seas, and one of the important reasons for the dead zones is the runoff of fertilizers from rivers, which affects the activity of microorganisms and depletes dissolved oxygen in oxygenated water, resulting in the creation of dead zones in coastal areas [45]. At the same time, excessive nitrogen fertilizer is not only harmful to the environment but also leads to an increase in internode length, a decrease in internode thickness, a decrease in bending resistance, and a significant increase in the number of collapses in rice stalks [6]. The localization study of N-uptake- and utilization-related genes in this study yielded several excellent genetic resources, which lays an important foundation for improving N fertilizer application and enhancing N uptake and utilization efficiency in the future.

N content in rice plants is usually represented in terms of concentration, and usually, the concentration is used to diagnose the plant’s nutritional adequacy, deficiency, or excess. Thus, the N concentration visually reflects the plant’s uptake of N [46]. The product of the weight of dry matter or grains and N content represents the amount of nutrient accumulation, which can be used as an indicator of N uptake and utilization and is directly related to crop yield [47]. N content and N accumulation in plants are closely related to N uptake and utilization [5]. For example, in order to study the effect of biochar on N uptake and utilization in rice, the total N uptake, internal N utilization efficiency, and grain yield in six seasons were measured, and the dry weight, N content, and N accumulation of straw, rachis, and leafy and leafless spikelets were measured in the evaluation of internal N uptake and utilization efficiency in rice plants [48]. In an experiment to investigate the relationship between the N use efficiency and yield of organic rice varieties, the N uptake and use efficiency of rice were assessed in terms of dry matter accumulation, N content, and N uptake (N accumulation), and the experimental conclusion was that the rice yield was significantly and positively correlated with N uptake [49].

N uptake and utilization efficiency are important research topics in rice cultivation worldwide [3,50]. In recent years, a lot of important genes related to N uptake and utilization in rice have been localized and identified by GWAS, such as *OsNLP4*, *OsNRT1.1B*, *OsNPF6.1*, *OsNAC42*, *OsTCP19*, etc. [22,23,24,51,52]. In this study, the N content and N accumulation in the roots and leaves of 295 *japonica* rice varieties at the seedling stage were measured under low- and high-N treatments, respectively. The above phenotypic data were used for GWAS to locate QTLs associated with N uptake and utilization in rice. The localized QTLs were mainly concentrated in linkage groups 4, 6, 8, 10, and 11, which is consistent with some previous studies [33,53,54]. Transcriptome analysis has been used to study the response of many plant species to various environmental stresses [55,56,57,58,59]. With the development of high-throughput sequencing technology and the continuous improvement of transcriptome databases, the combination of transcriptome data with traditional QTL mapping or GWAS has been widely used in the prediction of candidate genes. For example, by combining a population segregation analysis with transcriptomics, four candidate genes related to rice blast resistance were obtained [60]; by integrating GWAS and transcriptome data, one N utilization efficiency (NUE)-related candidate gene, *OsNAC68*, was identified in rice [61]. Our previous studies combining GWAS/BSA with transcriptome data identified many salinity tolerance candidate genes (*OsIRO3*, *OsSAP16*) [31,62]. In this study, we obtained 40 haplotypic differential genes within the interval localized by GWAS. By sequencing the transcriptomes of low-N-tolerant and low-N-sensitive varieties, five genes differentially expressed in low-N-tolerant varieties were identified as more valuable candidate genes. And according to the phenotypic differences between different haplotypes, five favorable alleles were identified, namely, Hap2 of *Os01g0159250*, Hap1 of *Os02g0618200*, Hap2 of *Os02g0618400*, Hap1 of *Os02g0630300*, and Hap2 of *Os06g0619000*. Varieties containing the above alleles showed excellent phenotypic variation. The study of these five genes in relation to N uptake and utilization in rice has not been reported. Varieties containing the above alleles exhibited excellent phenotypes, so the approach integrating GWAS and transcriptome data is an effective way to mine genes related to N uptake and utilization in rice.

Among the five most valuable candidate genes in this study, *Os02g0618200*, *Os02g0618400,* and *Os02g0630300* have been cloned. *Os02g0618200*, whose gene symbol is *OsPRR1*, encodes a response regulator that is expressed in multiple tissues of rice and has a circadian rhythm of expression in leaves, roots, tiller shoots, and endosperm. The expression of *OsPRR1* suppresses the expression of *OsCCA1*, which mediates rice tiller and spike development [41]. *Os02g0618400* (*OsMPS*) encodes an R2R3-like MYB transcription factor. *OsMPS* is expressed in many tissues, including the aboveground parts of seedlings, roots, pollen, vascular tissues of glumes, endosperm sheaths, and leaves, but not in the endosperm, stigma, ovary, or embryo; *OsMPS* can regulate adaptive growth by integrating signals between different plant hormones and the environment [42]. *Os02g0630300* (*OsGA2ox9*) encodes a gibberellin 2-oxidase. *OsGA2ox9* is transcribed in a variety of tissues, including rice roots, leaves, pollen, and seeds [40]. It was shown that *OsGA2ox9* knockout lines germinate spikes under continuous rainy weather, whereas seed dormancy was increased in overexpressing lines, but this was restored by externally applied GA3; *OsAmy* (α-amylase isozyme 3D) expression was significantly increased in *OsGA2ox9* knockout seeds, and the amount of glucose and sucrose in the seeds of the knockout lines was found to be increased; thus, *OsGA2ox9* may regulate the transcription of α-amylase-encoding genes through GA signaling, which promotes the hydrolysis of starch to soluble sugars and inhibits ABA signaling, leading to spike germination [40]. Two genes, *Os01g0159250* and *Os06g0619000*, were not cloned. *Os01g0159250* is a hypothetical protein. The expression product of *Os06g0619000* is a protein containing the structural domain of NB-ARC, and proteins containing the structural domain of NB-ARC are usually associated with plant adversity responses [63,64,65]. However, the responses of the five candidate genes to N have not been studied in depth. The two candidate genes that have not yet been cloned should be further investigated for their relevance to N uptake and utilization. And among the cloned genes, *Os02g0618200* (which mediates tillering and spike development), *Os02g0618400* (which regulates rice growth), and *Os02g0630300* (which inhibits ABA signaling) may be related to N regulation, and their molecular mechanisms in N uptake and utilization should be further investigated in depth in the future.

## 5. Conclusions

A total of 75 QTLs were obtained using GWAS, which contained 811 genes. Of the 811 genes, 281 showed different haplotypes, and 40 genes had significant phenotypic differences among different haplotypes. Five genes (*Os01g0159250*, *Os02g0618200*, *Os02g0618400*, *Os02g0630300*, and *Os06g0619000*) that differentially expressed in the low-N-tolerant variety (Longjing 31) were finally identified as the more valuable candidate genes associated with N uptake and utilization among these 40 genes. However, the molecular mechanisms involved in the regulation of N uptake and utilization by the above candidate genes still need to be further investigated.

## Figures and Tables

**Table 1:** The candidate genes that were expressed differentially under the low-N condition.

Candidate Genes	Regulated	QTLs	Annotation	Reference
*Os01g0159250*	up	*qLRNAA1*	Hypothetical protein	
*Os02g0630300*	down	*qLYNAA2*	2OG-Fe (II) oxygenase domain-containing protein	Xing et al., 2023 [40]
*Os02g0618200*	down	*qLRNAA2*	Circadian-associated rice pseudo-response regulator, control of flowering time	Wang et al., 2020 [41]
*Os02g0618400*	down	*qLRNAA2*	R2R3-type MYB transcription factor, adaptive growth regulation	Schmidt et al., 2013 [42]
*Os06g0619000*	down	*qRNAAR6-2*	NB-ARC domain-containing protein	

## Data Availability

The RNA-seq data for this study can be found in the National Center for Biotechnology Information under the accession number PRJNA835804 (https://www.ncbi.nlm.nih.gov/, accessed on 10 June 2023).

## Supplementary Materials

The following supporting information can be downloaded at https://www.mdpi.com/article/10.3390/genes15030327/s1: Table S1. Sequence of primers used in qRT-PCR; Table S2. Descriptive statistics of phenotypic traits in natural populations; Table S3. Phenotypic analysis of natural populations; Table S4. Significantly associated *loci*; Table S5. Annotation and haplotype analysis of associated genes; Table S6. RNA-seq data for 40 genes.

## Abbreviations

NUE: nitrogen use efficiency; GO: Gene Ontology; KEGG: Kyoto Encyclopedia of Genes and Genomes.
